# Pancreaticoduodenectomy versus limited resection for duodenal gastrointestinal stromal tumors: a systematic review and meta-analysis

**DOI:** 10.1186/s12893-019-0587-4

**Published:** 2019-08-28

**Authors:** Zefeng Shen, Ping Chen, Nannan Du, Parishit A. Khadaroo, Danyi Mao, Lihu Gu

**Affiliations:** 1 The Second Clinical Medical College, Zhejiang Chinese Medical University, Hangzhou, Zhejiang China; 2 Department of General Surgery, HwaMei Hospital, University of Chinese Academy of Sciences, Northwest Street 41, Haishu District, Ningbo, 315010 Zhejiang China; 3 Monash University School of Public Health and Preventive Medicine, Melbourne, Australia; 4 Basic Medical College, Zhejiang Chinese Medical University, Zhejiang, Hangzhou China

**Keywords:** Duodenal gastrointestinal stromal tumors, Pancreaticoduodenectomy, Limited resection, Prognosis, Meta-analysis

## Abstract

**Background:**

By comparing the long-term prognostic outcomes after pancreaticoduodenectomy (PD) and limited resection (LR), this study aimed to investigate the optimal surgical modality for duodenal gastrointestinal stromal tumors (GISTs).

**Methods:**

Two authors independently searched PubMed, Web of Science, Embase, and the Cochrane Library for published articles comparing the long-term prognostic and clinicopathological factors of duodenal GIST patients undergoing PD versus LR. Relevant information was extracted and analyzed.

**Results:**

After screening, 10 items comprising 623 cases were eventually included. This meta-analysis explicitly indicated that PD treatment was associated with worse long-term prognosis (hazard ratio = 1.93; 95% confidence interval [CI], 1.39–2.69; *p* < 0.001; I^2^ = 0) and more complications (odds ratio [OR] = 2.90; 95% CI, 1.90–4.42; *p* < 0.001; I^2^ = 10%) than LR treatment. Nevertheless, for duodenal GISTs, PD was related to the following clinicopathological features: invasion of the second part of the duodenum (OR = 3.39; 95% CI, 1.69–6.79; *p* < 0.001; I^2^ = 50%), high-degree tumor mitosis (> 5/50 high-power fields; OR = 2.24; 95% CI, 1.42–3.52; *p* < 0.001; I^2^ = 0), and high-risk classification (OR = 3.17; 95% CI; 2.13–4.71; *p* < 0.001; I^2^ = 0).

**Conclusions:**

Since PD is associated with worse long-term prognosis and more complications, its safety and efficacy should be ascertained. Our findings recommend the use of LR to obtain negative incision margins when conditions permit it.

**Electronic supplementary material:**

The online version of this article (10.1186/s12893-019-0587-4) contains supplementary material, which is available to authorized users.

## Background

Gastrointestinal stromal tumors (GISTs), with a global incidence of 11–19.6 per million people, are deemed the most frequently encountered mesenchymal tumors [1, 2]. GISTs can occur throughout the entire gastrointestinal tract from the esophagus to the rectum, but the primary positions of occurrence are the stomach (50–60%) and the small intestine (30–35%) [3–5]. Duodenal GISTs, despite accounting for only 3–5% of all GISTs, are generally differentiated from other small intestinal GISTs and highly concerning [6]. Diverse clinical manifestations, anatomical complexity of the pancreaticoduodenal region, and a lack of sufficient clinical experience due to low incidence make it challenging to diagnose, evaluate, and determine the optimal treatment strategy for GISTs [7, 8].

According to the results of relevant research, surgical excision without positive margins, tumor rupture, or spillover is the standard treatment for primary duodenal GISTs without metastasis [9, 10]. Despite surgical margins not being clearly defined, negative incision margins of 1–2 cm are recommended clinically [11]. Unlike adenocarcinomas, regional lymph node metastasis and vertical submucosal dissemination in GISTs are infrequent and circumscribed [12–14]. Even with the high recurrence risk of GISTs, the typical manifestation is limited to compression and displacement to the peripheral organs while lacking invasive potential [11]. Therefore, for primary GISTs without metastasis, lymph node dissection and enlarged resection cannot yield survival benefits [15]. Nevertheless, it should be emphasized that duodenal GISTs differ from those GISTs originating from other digestive tracts. The choice of surgical methods correlates with tumor size and invasiveness and is affected by whether they are adjacent to vital structures such as caput pancreatis, choledoch, hepatopancreatic ampulla, and mesentery root [6, 9].

Clinically adopted surgical excision modalities for duodenal GISTs include pancreaticoduodenectomy and limited resection (segmental or wedge-shaped duodenectomy) [16, 17], but the optimal surgical method remains controversial. Some studies demonstrated that compared with limited resection (LR), pancreaticoduodenectomy (PD) could achieve a wider margin, alleviating the hazard of positive margins and local recurrence [9, 11, 18]. Despite the possibility of recurrence, many surgeons still recommended the routine use of LR, which can preserve pancreatic function and gastrointestinal continuity, thereby reducing postoperative complications and accelerating the recovery of digestive capability [15, 19, 20]. However, the controversy reaches far beyond that. Some researchers recently indicated that rather than surgical modality, clinicopathological characteristics such as tumor size, mitosis degree, and National Institutes of Health (NIH) recurrence risk classification were the determinants of recurrence-free survival. When selecting PD or LR, surgeons consider elements such as tumor size and location, invasion or adhesion to adjacent organs, and overall patient fitness [21–23], which seem to be reasonable but make the determination of surgical modalities more ambiguous. The above studies all provided valuable clinical experience for the surgical treatment of duodenal GISTs, but the conclusions were biased and unpersuasive owing to insufficient sample size. Consequently, this meta-analysis included all studies comparing the prognostic outcomes after PD or LR to explore the optimal surgical modality for duodenal GISTs.

## Methods

### Bibliographic search

Referring to the gold standard, for which databases ought to be retrieved in surgical systematic reviews [24], two authors independently searched PubMed, Web of Science, Embase, and the Cochrane Library for published articles that compared the prognostic and clinicopathological factors of duodenal GIST patients undergoing PD or LR. Publication time of the included articles ranged from database inception until February 2019. Despite the existence of selection bias, to guarantee study quality and credibility, we limited our search to Scientific Citation Index papers. The search strategies included “duodenal gastrointestinal stromal tumor” OR “duodenal gastrointestinal stromal tumour” OR “gastrointestinal stromal tumor of duodenal” OR “gastrointestinal stromal tumor of duodenum.” This meta-analysis was implemented in accordance with the Preferred Reporting Items for Systematic Review and Meta-Analysis checklist published in 2009 [25] (Additional file 2: Table S1).

### Inclusion criteria

The inclusion criteria were as follows:

1. The clinicopathological data of all cases were complete, and all were pathologically diagnosed with duodenal GISTs;
2. All patients with duodenal GISTs were treated with PD or LR; and
3. Included articles provided hazard ratios (HRs) and 95% confidence intervals (CIs) to compare the long-term prognosis of the two surgical modalities (PD versus LR).

The exclusion criteria were as follows:

1. Case reports, guidelines, expert consensus, histopathological studies, or literature reviews;
2. Failure to provide sufficient data to compare the long-term prognosis of the two surgical modalities (PD versus LR); and
3. Failure to provide separate clinicopathological data for PD or LR.

### Data extraction and quality evaluation

Using the inclusion and exclusion criteria, two researchers independently screened the retrieved articles and extracted the relevant information. In cases of disagreement, we obtained the final results through discussion or third-party arbitration. For articles lacking the indispensable information, we attempted to contact the original authors to ensure study integrity. Extracted data included: author, year of publication, country, patients’ baseline and clinicopathological information, postoperative complications, usage of imatinib, follow-up time, and HR for comparing the long-term prognosis differences between the two surgical modalities (PD versus LR).

The methodological quality of each included retrospective cohort studies was assessed by the Newcastle-Ottawa Scale (NOS). Individually, the included articles were evaluated for three aspects: object selection, inter-group comparability, and outcome measurement. Materials with scores < 6 were considered of low quality [26].

### Statistical analysis

Using Revman 5.3 software, we combined the HR and 95% CI from each study to quantificationally analyze the long-term prognostic differences between the two surgical modalities; we used pooled odds ratio (OR) and 95% CI values to compare the clinicopathological distribution distinction between the two groups. Thereafter, the clinical, methodological, and statistical heterogeneity of the included articles was assessed judiciously to determine the application of random-effect or fixed-effect models.

Stata 12.0 software was used to evaluate study sensitivity and publication bias. Publication bias could be quantified by Egger’s test, in which values of *p* < 0.05 were considered statistically significant. However, Egger’s test of publication bias was not performed on the analysis subgroup with fewer than 10 studies because of the low sensitivity of the qualitative and quantitative tests [27].

## Results

### Data collection and characteristics

**Fig. 1:**
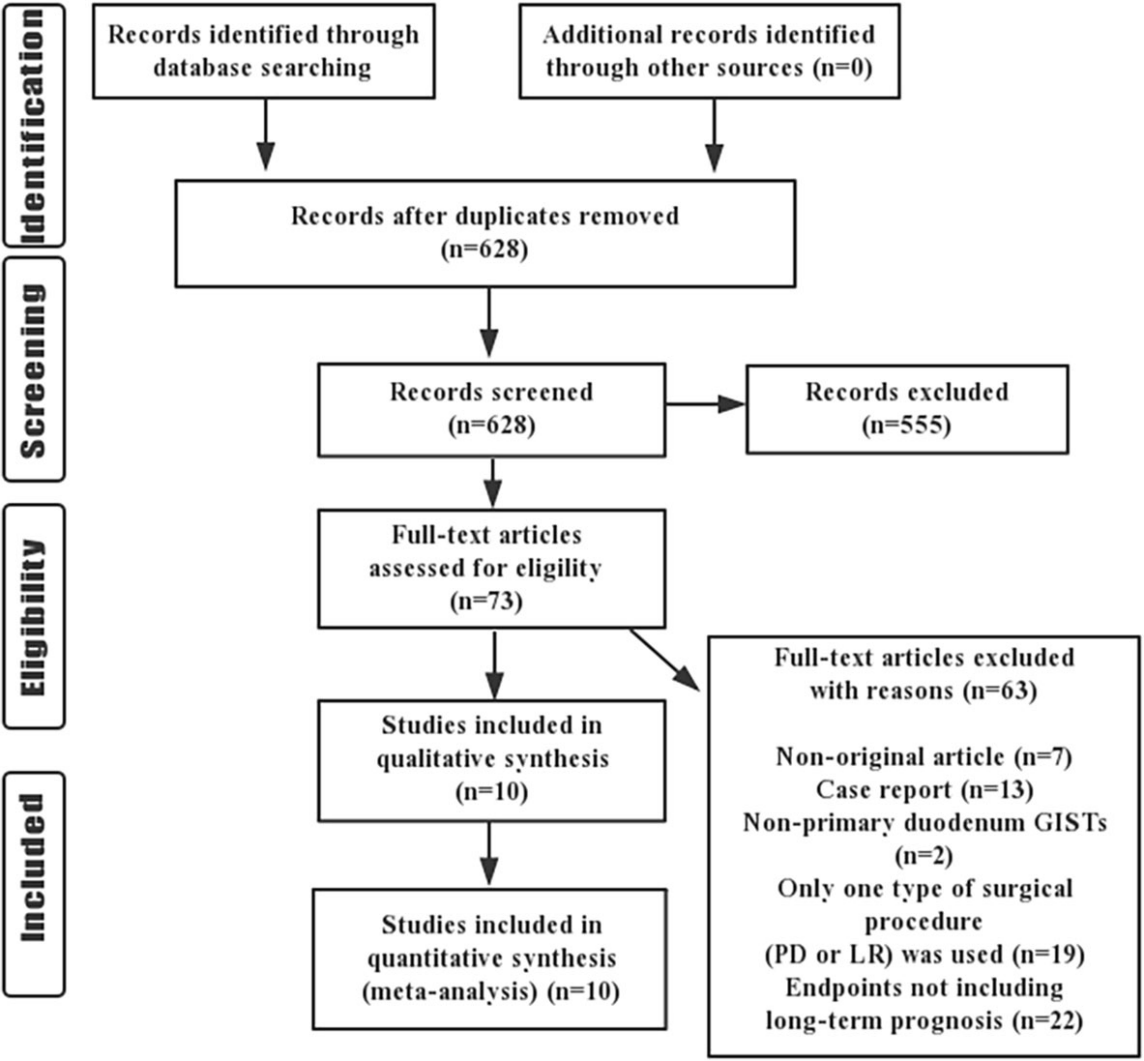
Flowchart of literature screening process

A total of 627 relevant articles were initially retrieved. After the screening, 10 items comprising 623 cases were eventually determined; the reasons for the exclusion of other materials are summarized in Fig. 1. The included retrospective cohort studies consisting of six single-center and four multi-center studies originated in China, Korea, Japan, France, Italy, Germany, Poland, and the United States; all were published in English. There were 25–105 patients in each study, for a total of 623 patients enduring PD or LR resection for primary duodenal GISTs. Patient characteristics and methodological quality evaluation data are displayed in Table 1.

**Table 1:** Summary of 10 studies reporting surgical outcomes of duodenal gastrointestinal stromal tumors (GISTs)

Author, year	Country	No.	Location(D2)	Mitosis (/50HPF)		Risk category		Margins resection		Postoperative complication	Imatinib therapy	Median follow-up time
				≥5	< 5	High/Intermediate	Low/Very low	R0	R1/R2			
Gu [28], 2018	China	T: 62	T: 32	T: 10	T: 52	T: 28	T: 34	T: 61	T: 1	T: 11	T: 12	> 33 months
		LR: 47	LR: 18	LR: 6	LR: 41	LR: 20	LR: 27	LR: 46	LR: 1	LR: 4	LR: 10	
		PD: 15	PD: 14	PD: 4	PD: 11	PD: 8	PD: 7	PD: 15	PD: 0	PD: 7	PD: 2	
Lee, [21] 2018	South Korea	T: 98	T: 61	T: 25	T: 73	T: 35	T: 63	T: 95	T: 3	T: 32	T: 40	46 months
		LR: 53	LR: NA	LR: 8	LR: 45	LR: 11	LR: 42	LR: 50	LR: 3	LR: 15	LR: NA	
		PD: 45	PD: NA	PD: 17	PD: 28	PD: 24	PD: 21	PD: 45	PD: 0	PD:17	PD: NA	
Zhang, [22] 2018	China	T: 51	T: 33	T: 18	T: 30	T: 19	T: 32	T: 47	T: 4	T: 7	T: 23	36 months
		LR: 37	LR: 24	LR: 13	LR: 21	LR: 12	LR: 25	LR: 33	LR: 4	LR: 4	LR: NA	
		PD: 14	PD: 9	PD: 5	PD: 9	PD: 7	PD: 7	PD: 14	PD: 0	PD: 3	PD: NA	
Shi, [19] 2017	China	T: 61	T: 33	T: 10	T: 51	T: 24	T: 37	All R0		T: 24	T: 13	> 5 years
		LR: 45	LR: 22	LR: 6	LR: 39	LR:15	LR:30			LR:15	LR: NA	
		PD: 16	PD: 11	PD: 4	PD: 12	PD:9	PD:7			PD:9	PD: NA	
Chen, [23] 2017	China	T: 64	T: 30	T: 22	T: 42	T: 33	T: 31	All R0		T: 29	T: 15	> 5 years
		LR: 41	LR: 18	LR: 12	LR: 29	LR: 17	LR: 24			LR: 13	LR: 8	
		PD: 23	PD: 12	PD: 10	PD: 13	PD: 16	PD: 7			PD: 16	PD: 7	
Sugase, [20] 2016	Japan	T: 25	T: 14	NA	NA	T: 9	T: 16	All R0		T: 8	T: 3	> 5 years
		LR: 16	LR: 7			LR: 3	LR: 13			LR: 5	LR: 1	
		PD: 9	PD: 7			PD: 6	PD: 3			PD: 3	PD: 2	
Duffaud, [29] 2014	France	T: 105	T: 38	NA	NA	NA	NA	T: 85	T: 13	T: 21	T: 26	36 months
		LR: 82	LR: NA					LR: 68	LR: 10	LR: 15	LR: NA	
		PD: 23	PD: NA					PD: 17	PD: 3	PD: 6	PD: NA	
Zhou, [30] 2013	China	T: 48	T: 17	T: 12	T: 36	T: 20	T: 28	All R0		T: 9	T: 9	36 months
		LR: 34	LR: 8	LR: NA	LR: NA	LR: 11	LR: 23			LR: 4	LR: NA	
		PD: 14	PD: 9	PD: NA	PD: NA	PD: 9	PD: 5			PD: 5	PD: NA	
Colombo, [15] 2012	Italy,	T: 84	T: 21	T: 22	T: 56	T: 43	T: 41	T: 45	T: 9	T: 15	T: 34	42 months
		LR: 56	LR: 6	LR: 11	LR: 41	LR: 24	LR: 32	LR: 26	LR: 9	LR: 5	LR: 19	
		PD: 28	PD: 15	PD: 11	PD: 15	PD: 19	PD: 9	PD: 19	PD: 0	PD: 10	PD:15	
Tien, [31] 2010	China	T: 25	T: 13	T: 8	T: 17	T: 14	T: 11	T: 24	T: 1	T: 6	NOT	18 months
		LR: 16	LR: 7	LR: 4	LR: 12	LR: 6	LR: 10	LR: 15	LR: 1	LR: 2		
		PD: 9	PD: 6	PD: 4	PD: 5	PD: 8	PD: 1	PD: 9	PD: 0	PD: 4		

*LR* Limited resection, *PD* Pancreaticoduodenectomy, *T* total, *NA* Not available, *NOT* No patients underwent imatinib therapy, *D2* The second part of the duodenum, *R0* Complete excision with all margins negative, *R1* Incomplete excision with positive margins under microscopy, *R2* Incomplete excision with macroscopic positive margins

The NOS was used to evaluate the quality of the included articles, and the results demonstrated that all were of high quality (Table 2). Owing to the low incidence of duodenal GISTs, researchers seldom implemented studies in the allied domains, except for retrospective studies whose clinical heterogeneity was remarkable but inevitable; thus, the random-effects models were routinely adopted.

**Table 2:** Newcastle-Ottawa scale as a quality assessment

Author, year	Selection				Comparability	Outcome			Total score
	Exposed cohort	Non-exposed cohort	Ascertainment of exposure	Outcome of interest	Control for factor	Assessment of outcome	Follow-up long enough	Adequacy of follow-up	
Gu [28], 2018	*	*	*	*	*		*	*	7
Lee, [21] 2018	*	*	*	*	*		*	*	7
Zhang, [22] 2018	*	*	*		*		*	*	6
Shi, [19] 2017	*	*	*	*	*		*	*	7
Chen, [23] 2017	*	*	*	*	*		*	*	7
Sugase, [20] 2016	*	*	*	*	*		*	*	7
Duffaud, [29] 2014	*	*	*	*	*		*	*	7
Zhou, [30] 2013	*	*	*	*	*		*	*	7
Colombo, [15] 2012	*	*	*	*	*		*	*	7
Tien, [31] 2010	*	*	*	*	*		*		6

### Prognostic factors of long-term outcomes

**Fig. 2:**
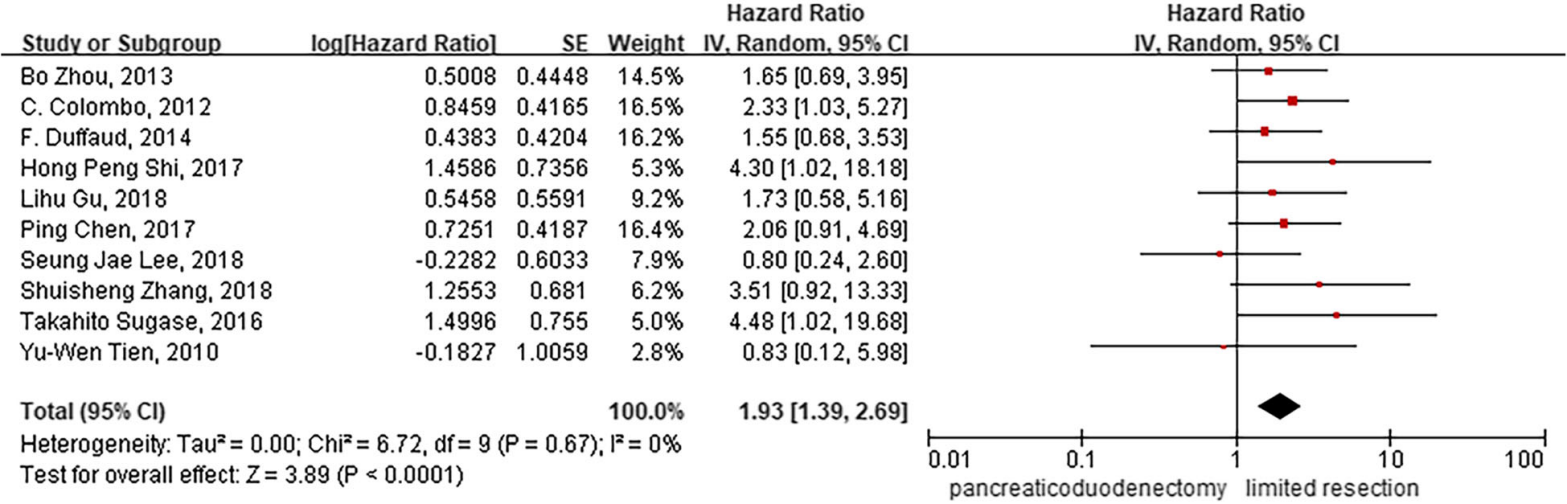
Forest plot comparing the long-term prognostic outcomes of pancreaticoduodenectomy (PD) and limited resection (LR)

Of the 623 patients in the analysis, 196 underwent PD treatment and 427 underwent LR. In this meta-analysis, the relationship between the long-term prognosis of duodenal GISTs and surgical modalities was evaluated. The outcome revealed that PD treatment was associated with worse long-term prognosis than LR (HR = 1.93; 95% CI, 1.39–2.69; *p* < 0.001; I^2^ = 0; Table 3, Fig. 2).

**Table 3:** Combination of hazard ratio (HR) associated with recurrence-free survival of duodenal gastrointestinal stromal tumors

	No. of studies	No. of patients	HR	95%CI	*p* value	Heterogeneity (I^2^), %	Egger’s test (*p* value)
Gender (Male vs. Female)	8	500	1.42	1.02–1.97	**0.04**	0	–
Age (≥60 vs. < 60)	5	343	1.12	0.72–1.77	0.61	0	–
Tumor size (≥5 vs. < 5 cm)	8	550	3.59	2.32–5.55	**< 0.001**	26	–
No. of mitosis/50 HPF (≥5 vs. < 5)	8	550	4.10	2.11–7.98	**< 0.001**	62	–
Type of surgery (PD vs. LR)	10	623	1.93	1.39–2.69	**< 0.001**	0	0.639
Imatinib treatment (Yes vs. no)	7	525	0.95	0.60–1.49	0.82	0	–
Risk grade (High/Other)	3	135	6.33	2.04–19.66	**0.001**	33	–

Apart from investigating the impact of surgical modalities on long-term tumor prognosis, we also found that male patients were prone to relapse (HR = 1.42; 95% CI, 1.02–1.97; *p* = 0.04; I^2^ = 0). Tremendous tumors (> 5 cm) (HR = 3.59; 95% CI, 2.32–5.55; *p* < 0.001; I^2^ = 26%), high-degree tumor mitosis (> 5/50 high-power fields [HPF]) (HR = 4.10; 95% CI, 2.11–7.98; *p* < 0.001; I^2^ = 62%), and high-risk classification (HR = 6.33; 95% CI, 2.04–19.66; *p =* 0.001; I^2^ = 33%) frequently implied a more unsatisfactory long-term prognosis of patients with duodenal GISTs. Furthermore, imatinib, the adjuvant drug commonly used to treat GISTs, did not decrease recrudescence in this meta-analysis (HR = 0.95; 95% CI, 0.60–1.49; *p* = 0.82; I^2^ = 0) (Table 3).

### Clinicopathological characteristics by surgical group

The distribution of the clinicopathological features by surgical group is shown in Table 4. The patients exhibiting the following clinicopathological characteristics were more likely to undergo PD: invasion of the second part of the duodenum (OR = 3.39; 95% CI, 1.69–6.79; *p* < 0.001; I^2^ = 50%), high-degree tumor mitosis (> 5/50 HPF) (OR = 2.24, 95% CI, 1.42–3.52; *p* < 0.001; I^2^ = 0), and high-risk classifications (OR = 3.17, 95% CI, 2.13–4.71; *p* < 0.001; I^2^ = 0). The combined OR value also indicated that the short-term prognosis (postoperative complications) in the PD group was worse than that in the LR group (OR = 2.90; 95% CI, 1.90–4.42; *p* < 0.001; I^2^ = 10%). Nevertheless, there were no significant intergroup differences in incision margin (OR = 0.54; 95% CI, 0.20–1.45; *p* = 0.22; I^2^ = 0), imatinib usage (OR = 1.78; 95% CI, 0.93–3.38; *p* = 0.08; I^2^ = 0), and sex distribution (OR = 1.38; 95% CI, 0.95–2.02; *p* = 0.09; I^2^ = 0; Table 4).

**Table 4:** Distribution of clinicopathological characteristics by surgical group

	No. of studies	PD group		LR group		OR	95%CI	*p* value	Heterogeneity (I^2^), %	Egger’s test (*p* value)
		Total	Events	Total	Events					
Gender (male)	9	173	102	345	173	1.38	0.95–2.02	0.09	0	–
Location(D2)	8	128	83	292	110	3.39	1.69–6.79	**< 0.001**	50	–
Mitotic count ≥5	7	148	55	288	60	2.24	1.42–3.52	**< 0.001**	0	–
Risk category (High+Intermediate)	9	173	106	345	119	3.17	2.13–4.71	**< 0.001**	0	–
Margins resection (R1 + R2)	10	184	3	402	28	0.54	0.20–1.45	0.22	0	0.181
Postoperative complication	10	196	80	427	82	2.90	1.90–4.42	**< 0.001**	10	0.319
Imatinib therapy	5	84	26	176	38	1.78	0.93–3.38	0.08	0	–

*LR* Limited resection, *PD* Pancreaticoduodenectomy, *D2* The second part of the duodenum, *OR* Odds ratio, *CI* Confidence interval, *R1* Incomplete excision with positive margins under microscopy, *R2* Incomplete excision with macroscopic positive margins

### Publication bias and sensitivity analyses

**Fig. 3:**
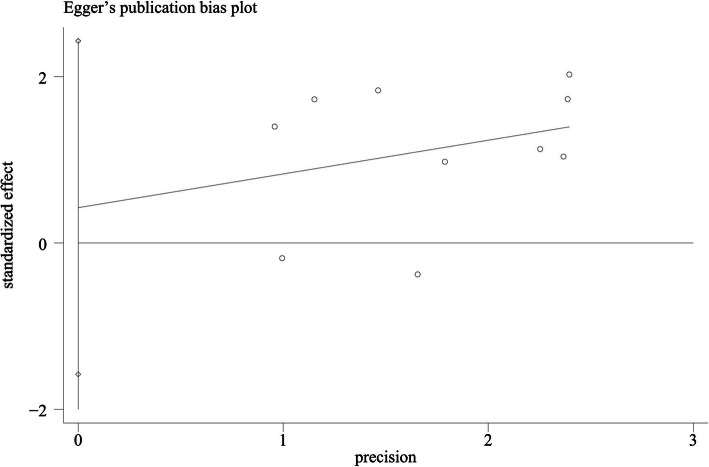
Egger’s funnel plot for publication bias test comparing the curative effects of the different surgical groups

Egger′s test (*p* = 0.639) certified the lack of publication bias in these 10 articles on the relationship between long-term prognosis and surgical modalities (Fig. 3). Other test outcomes are recorded in Tables 3 and 4. Beyond that, the sensitivity analysis manifested the excellent stability of the above conclusions (Additional file 1: Figure S1).

## Discussion

For non-metastatic primary duodenal GISTs, local resection with negative incision margins is a potential therapeutic option. However, considering the anatomical complexity of the pancreaticoduodenal region, establishing the optimal treatment strategy remains challenging. To resolve this controversy, researchers from Singapore published a meta-analysis of relevant studies in 2014 [32]. Despite specific referential significance, the few existing clinical studies that met the requirements before 2014, and the authors’ inappropriate methodology in the systematic evaluation, its limitations were apparent. When charting disease-free survival–related forest maps, they included seven studies comprising 260 patients in the meta-analysis, of which three did not provide HR values. The authors did not exclude the three articles lacking data but directly utilized the total number of relapses during follow-up to derive the HR values related to disease-free survival, without considering the influence of follow-up time on the outcomes. The authors’ methodological errors were also reflected in the misused risk ratio, an index for evaluating prospective studies, to assess the retrospective studies while plotting the forest maps associated with overall complication rates and overall disease recurrence, whose conclusions lacked credibility. To make the consequences more evidence-based and compensate for the deficiency of the predecessors, we cautiously included all articles that could provide sufficient data to compare the long-term prognosis of duodenal GISTs after PD or LR according to the inclusion and exclusion criteria. Furthermore, we also explored the distribution of the tumors’ clinicopathological features in the different surgical groups to strengthen the conclusion.

The findings of this meta-analysis explicitly indicated that PD treatment was associated with worse long-term prognosis and a higher tumor recurrence rate. Simultaneously, we found this conclusion credible since we proved the statistical homogeneity of the included articles measured by the heterogeneity test, non-existence of publication bias analyzed by Egger’s test, and stability of the results detected by the sensitivity analysis. As one of the most complicated abdominal surgeries, PD is associated with a higher incidence of postoperative complications, which is probably attributable to the severe trauma caused by multi-organ excision [33–35]. Among them, pancreatic and biliary fistulas, abdominal infection, and hemorrhage are frequent and serious complications after PD. Nevertheless, this did not directly demonstrate that the PD was a failure and should be prohibited, nor did it suggest that its clinical usage equated to overtreatment. Depending on the collection and analysis of retrospective cohorts, this meta-analysis could merely indicate a correlation between PD and worse long-term prognosis rather than a causal relationship. It should be stressed that the selection of PD is related to the following clinicopathological features: invasion of the second part of the duodenum, high-degree tumor mitosis (> 5/50 HPF), and high-risk classifications. This indicates why PD was associated with worse long-term prognosis, partially explains why PD is related to a higher malignancy rate, and demonstrates the higher recurrence potential of the duodenal GISTs treated with PD. To further explore PD efficacy and safety, researchers must complete more prospective studies that compare the treatment effects of the same high-risk duodenal GISTs after PD or LR. In conclusion, PD selection should be made carefully, and the use of LR is recommended to obtain negative incision margins when conditions permit it.

Consistent with NIH standards [36], this meta-analysis indicated that large tumors (> 5 cm), high-degree tumor mitosis (> 5/50 HPF), and high-risk classifications were correlated with a more unsatisfactory long-term prognosis. As the subgroup analysis showed, males tended to have a worse long-term prognosis, suggesting that sex hormones and their receptors might affect the progression of duodenal GISTs. Some small sample studies adopting immunohistochemistry discovered the negative expression of estrogen and progesterone receptors in duodenal GISTs [37, 38]. Tumors expressing androgen receptors are more prone to result in extraintestinal metastasis and be evaluated as high-risk [39]. Thus, the effect of sex on the long-term prognosis of tumors deserved further investigation, and their results would provide a theoretical basis for the use of sex hormone deprivation therapy in the treatment of duodenal GISTs.

Imatinib is a micromolecular tyrosine kinase inhibitor (TKI) that can antagonize the activities of KIT, PDGFR, and ABL kinase and is the first TKI approved by the United States Food and Drug Administration for the treatment of metastatic or unresectable GISTs. However, the results of this meta-analysis did not demonstrate the ability of imatinib to prevent recurrence. One of the reasons for this outcome could be that the patients failed to consistently take the prescribed imatinib after the surgical intervention. Correlative studies proposed that clinical surgeons consider 3-year continuous use of imatinib after resection as a criterion for the treatment of GISTs with a high recurrence risk [15, 29]. Otherwise, the tumors will continue to progress. Moreover, it should not be overestimated that the efficacy of imatinib targeted therapy for GISTs is related to tumor genotyping. Common mutation sites include exons 9, 11, 13, and 17 of the *c-kit* gene and exons 12, 14, and 18 of the *PDGFRα* gene. Among them, duodenal GISTs with mutation of exon 11 of the *c-kit* gene is most sensitive to imatinib [40, 41]. While, in accordance with the experiments of Corless et al. [42], tumors with the exon 18 mutation exhibit resistance. Before executing imatinib-targeted therapy, researchers should perform gene detection projects. However, the majority of the included clinical studies did not detect the mutations in the related genes; thus, the blind use of imatinib could not achieve the anticipated outcomes.

## Limitations

First, although all the included articles were of high quality in accordance with the NOS, the selection and recall biases of these retrospective studies should be considered. Second, this meta-analysis demonstrated a correlation rather than causality between PD treatment and worse long-term prognosis of GISTs but did not confirm that PD is a failed surgical procedure that should be abolished. Furthermore, the influence of race on tumor progression could not be neglected; eight of 10 included articles originated in Asian countries, implying a lack of research in non-Asian countries, which to some extent engenders publication bias and limits the generalizability of our results.

## Conclusions

This meta-analysis extracted the relevant information of the included articles and demonstrated that PD treatment was associated with worse long-term prognosis and more complications than LR. However, the results did not directly certify that PD was a manifestation of overtreatment because this consequence could be partially attributed to the greater malignancy and recurrence potential of the duodenal GISTs treated with PD.

## Additional files

Additional file 1: **Figure S1.** Sensitivity analysis including comparison of curative effects by surgical group. (TIF 9619 kb)

Additional file 2: **Table S1.** Preferred Reporting Items for Systematic Review and Meta-Analysis 2009 checklist. (DOC 54 kb)

## Data Availability

The datasets supporting the conclusions of this article are included within the article and its additional files.

## Abbreviations

CI: Confidence interval
GISTs: Gastrointestinal stromal tumors
HR: Hazard ratio
LR: Limited resection
NOS: Newcastle-Ottawa Scale
OR: Odds ratio
PD: Pancreaticoduodenectomy
TKI: Tyrosine kinase inhibitor
