# Supplementary figures and images for: Pancreaticoduodenectomy versus limited resection for duodenal gastrointestinal stromal tumors: a systematic review and meta-analysis
Source: BMC Surg. 2019 Aug 28;19:121. doi: 10.1186/s12893-019-0587-4 (PMC6712818; doi:10.1186/s12893-019-0587-4)

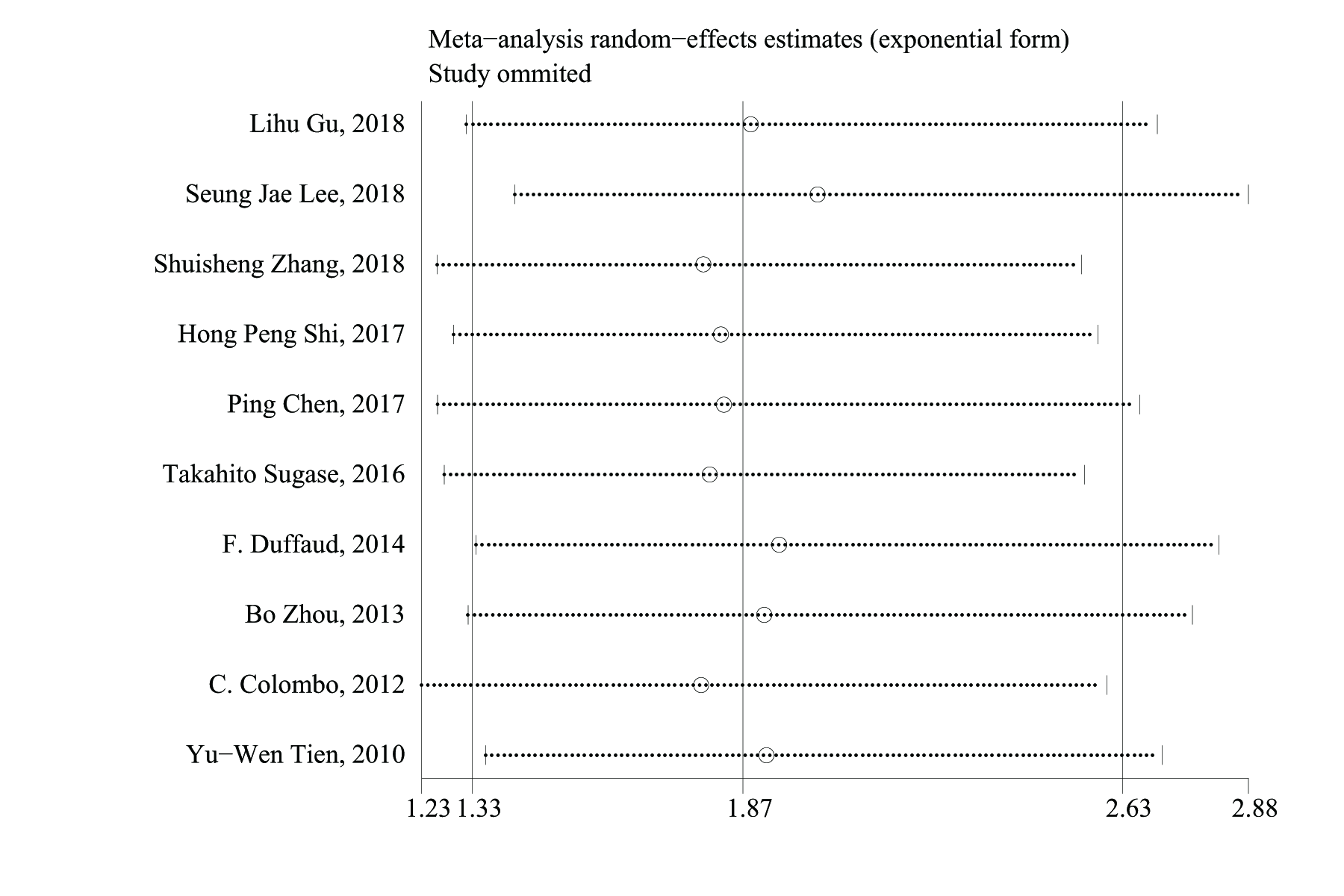

Supplement: Supplementary file 1 — Figure S1. Sensitivity analysis including comparison of curative effects by surgical group. (TIF 9619 kb) [file 12893_2019_587_MOESM1_ESM.tif]
